# Identification of a Gene Encoding Slow Skeletal Muscle Troponin T as a Novel Marker for Immortalization of Retinal Pigment Epithelial Cells

**DOI:** 10.1038/s41598-017-08014-w

**Published:** 2017-08-15

**Authors:** Takuya Kuroda, Satoshi Yasuda, Hiroyuki Nakashima, Nozomi Takada, Satoko Matsuyama, Shinji Kusakawa, Akihiro Umezawa, Akifumi Matsuyama, Shin Kawamata, Yoji Sato

**Affiliations:** 1 Division of Cell-Based Therapeutic Products, National Institute of Health Sciences, Tokyo, Japan; 2 Foundation for Biomedical Research and Innovation, Kobe, Japan; 3 Department of Reproductive Biology, National Research Institute for Child Health and Development, Tokyo, Japan; 4 Platform of Therapeutics for Rare Disease, National Institutes of Biomedical Innovation, Health and Nutrition, Osaka, Japan; 5 Department of Quality Assurance Science for Pharmaceuticals, Graduate School of Pharmaceutical Sciences, Nagoya City University, Nagoya, Japan; 6 Department of Cellular & Gene Therapy Products, Graduate School of Pharmaceutical Sciences, Osaka University, Osaka, Japan; 7 Department of Drug Discovery and Evolution, Graduated School of Pharmaceutical Sciences, Kyushu University, Fukuoka, Japan

## Abstract

Human pluripotent stem cells (hPSCs) are leading candidate raw materials for cell-based therapeutic products (CTPs). In the development of hPSC-derived CTPs, it is imperative to ensure that they do not form tumors after transplantation for safety reasons. Because cellular immortalization is a landmark of malignant transformation and a common feature of cancer cells, we aimed to develop an *in vitro* assay for detecting immortalized cells in CTPs. We employed retinal pigment epithelial (RPE) cells as a model of hPSC-derived products and identified a gene encoding slow skeletal muscle troponin T (*TNNT1*) as a novel marker of immortalized RPE cells by comprehensive microarray analysis. *TNNT1* mRNA was commonly upregulated in immortalized RPE cells and human induced pluripotent stem cells (hiPSCs), which have self-renewal ability. Additionally, we demonstrated that *TNNT1* mRNA expression is higher in several cancer tissues than in normal tissues. Furthermore, stable expression of *TNNT1* in ARPE-19 cells affected actin filament organization and enhanced their migration ability. Finally, we established a simple and rapid qRT-PCR assay targeting *TNNT1* transcripts that detected as low as 3% of ARPE-19 cells contained in normal primary RPE cells. Purified hiPSC-derived RPE cells showed *TNNT1* expression levels below the detection limit determined with primary RPE cells. Our qRT-PCR method is expected to greatly contribute to process validation and quality control of CTPs.

## Introduction

Human embryonic stem cells (hESCs)^1^ and human induced pluripotent stem cells (hiPSCs)^2^ are regarded promising cell sources for transplantation in regenerative medicine. The challenges associated with the complex stem cell-derived products used in regenerative medicine require great scientific progress. In addition to the dynamic complexity of their biology, several safety concerns for human pluripotent stem cell (hPSC)-derived products have hindered their clinical translation, including the genomic instability of hPSCs and the risk of tumorigenicity^3, 4^.

The ability to confirm the quality and safety of cell-based therapeutic products (CTPs) in the manufacturing process will be a critical factor in the anticipated success of regenerative medicine. One of the most important issues in the development of safe hPSC-derived CTPs is ensuring that the final product does not form tumors after implantation^3^. There are two main causes of tumor formation from hPSC-derived CTPs. Firstly, products derived from hPSCs might contain residual undifferentiated stem cells that might proliferate and form teratomas^5^. Secondly, some cells in the products may transform to finally form tumors. The latter is a common issue in CTPs, regardless of the cell type used as raw material^6^.

To address the issue of tumorigenicity, recent publications have advocated the development of highly efficient differentiation protocols for hPSCs^7–9^ and have outlined methods for eliminating residual hPSCs in the products^10, 11^. Several methods for detecting a small population of residual undifferentiated cells in hPSC-derived CTPs have been reported: (1) *in vivo* tumorigenicity tests that detect teratoma formation in severe immunodeficient NOG mice^12^, (2) detection of *LIN28* mRNA as an undifferentiated cell marker using quantitative reverse transcription (qRT-)PCR and droplet digital PCR^13, 14^, and (3) a highly efficient culture method for residual undifferentiated hiPSCs in products^15^. Similarly, assay methods have been developed for the detection of small numbers of transformed cells in CTPs: (1) *in vivo* tumorigenicity tests with NOG mice^16^, (2) digital analysis of soft agar colony formation^17^, and (3) cell growth analysis^18^. Although these assays are sensitive, they are time-consuming.

Cellular immortalization is widely known as a key step in the development of most human cancers, a defining property of cancer cells, and a prerequisite for cell transformation^19^. Therefore, we attempted to seek a novel immortalized cell marker and develop a rapid assay for detecting immortalized cells contained in CTPs. In this study, we employed retinal pigment epithelial (RPE) cells as a model of hPSC-derived CTPs because of their wide use in hPSC-derived CTPs. In the case of immortalization in primary human RPE cells, four papers have reported the establishment of immortalized RPE cell lines that spontaneously arose during *in vitro* culture^20–23^. Thus, it cannot be denied that immortalized RPE cells appear in products during the manufacturing process. Comprehensive microarray-based gene expression analysis indicated a marked upregulation of 15 transcripts in immortalized RPE cells. Our study identified a gene encoding slow skeletal muscle troponin T (*TNNT1*) as a suitable marker of immortalized RPE cells contained in hPSC-derived CTPs, and we propose a qRT-PCR method for the detection of this marker.

## Results

### Detection of Immortalized RPE Cells with Conventional Assays

**Figure 1:**
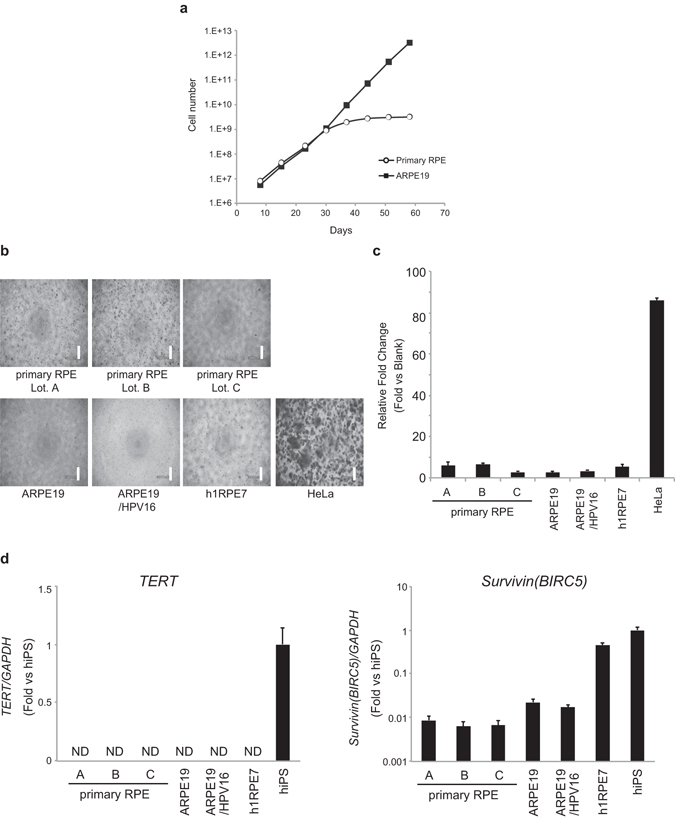
Immortalized RPE cells are undetectable by conventional *in vitro* assays. (**a**) Cell growth analysis of primary RPE cells and ARPE-19 cells. (**b**,**c**) A soft agar colony formation assay was carried out to detect immortalized RPE cells. Phase-contrast images of primary RPE cells, immortalized RPE cells, and HeLa cells cultured in soft agar medium for 30 days (b; Scale bars, 300 µm). Quantification of the cellular DNA is shown in a bar graph. Results were expressed as a relative fold change of the value of blank well. Data are presented as the mean ± standard deviation of three independent experiments (c). **(d)** The relative mRNA expression of *TERT* and *BIRC5*/Survivin in primary RPE cells, immortalized RPE cells, and hiPSCs was determined by qRT-PCR. Data are presented as the mean ± standard deviation of three independent experiments. N.D.: not detected.

To develop a detection assay of immortalized cells contaminating hPSC-derived RPE cells, we used three commercial immortalized RPE cell lines, and three lots of primary RPE cells as non-immortalized cell control samples. The following three immortalized RPE cell lines were used: ARPE-19, a spontaneously immortalized cell line^21^; ARPE-19/HPV-16, an ARPE-19 cell line transfected with DH-5 HPV-16; h1RPE7, a cell line derived from primary RPE cells transfected with a SV40 large T antigen^24^. Growth curve analysis confirmed that the growth rate of the primary RPE cells decreased as passage progressed, whereas ARPE-19 cells continued to proliferate (Fig. 1a). First, we sought to assess whether conventional assays were able to detect the immortalized RPE cells. This assay is generally used to evaluate cell transformation *in vitro*, demonstrated by the ability of the cells to grow in an anchorage-independent environment^25^. To quantify cell transformation, we used the CytoSelect 96-well Cell Transformation Assay. Unlike HeLa cells, which were used as a positive control, all immortalized RPE cell lines as well as primary RPE cells failed to grow in soft agar media when seeded at a density of 1.0 × 10^4^ cells/well (Fig. 1b,c). These results demonstrated that the soft agar colony formation assay is not appropriate for the detection of immortalized RPE cells.

Several tumor markers have shown to be detected with varying degrees of sensitivity and specificity^26, 27^. Accordingly, we expected that qRT-PCR detection of the tumor markers *TERT* and *Survivin/BIRC5* would contribute to a sensitive assay of immortalized RPE cells. Contrary to our expectation, *TERT* was not detected in all of the immortalized RPE cell lines, while it was detected in hiPSCs (Fig. 1d). On the other hand, *Survivin/BIRC5* was highly expressed in h1RPE7 cells and hiPSCs but not in ARPE-19 or ARPE-19/HPV-16 cells as compared to primary RPE cells. Therefore, *TERT* and *Survivin/BIRC5* are not likely to be suitable marker genes for immortalized RPE cells.

### Identification of Marker Genes for Immortalized RPE Cells

**Figure 2:**
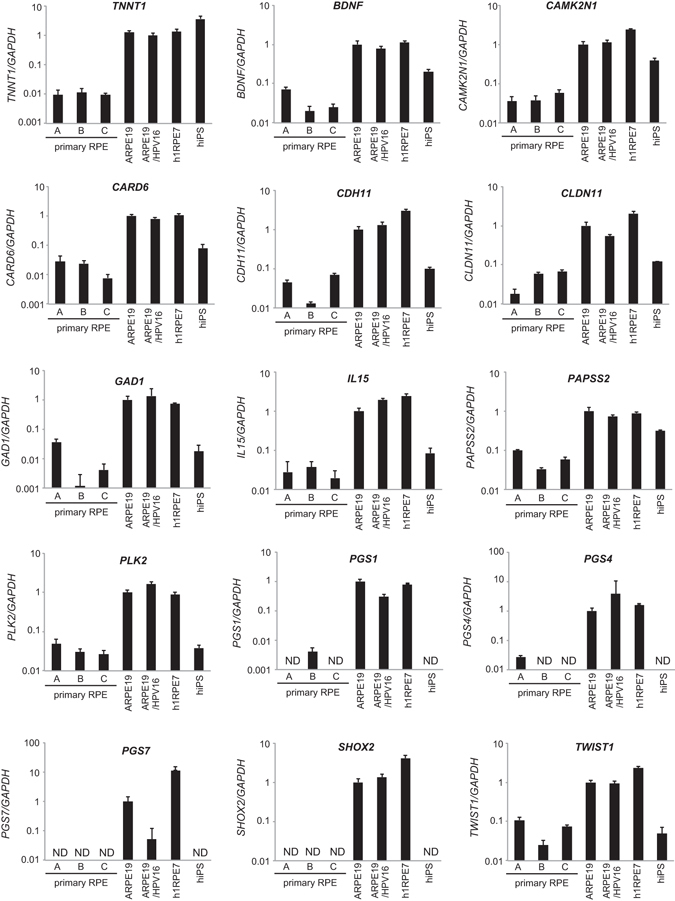
Identification of immortalized RPE cell marker genes. qRT-PCR analysis of immortalized RPE cell marker candidate genes in primary RPE cells, immortalized RPE cells, and hiPSCs. Bar graph represents fold gene expression relative to ARPE-19 cells. Results are means ± standard deviations (n = 5). N.D.: not detected.

We attempted to identify a novel marker gene of immortalized RPE cells. To this end, we compared the transcription profiles of the three lots of primary RPE cells and three immortalized RPE cell lines using microarray analysis. In total, 15 genes [*BDNF* (brain-derived neurotrophic factor), *CAMK2N1* (calcium/calmodulin-dependent protein kinase II inhibitor 1), *CARD6* (caspase recruitment domain family, member 6), *CDH11* (cadherin 11), *CLDN11* (claudin 11), *GAD1* (glutamate decarboxylase 1), *IL15* (interleukin 15), *PAPSS2* (3′-phosphoadenosine 5′-phosphosulfate synthase 2), *PLK2* (polo-like kinase 2), *PSG1* (pregnancy specific beta-1-glycoprotein 1), *PSG4* (pregnancy-specific beta-1-glycoprotein 4), *PSG7* (pregnancy-specific beta-1-glycoprotein 7), *SHOX2* (short stature homeobox 2), *TNNT1* (troponin T type 1), *TWIST1* (twist homolog 1)] were highly expressed in immortalized versus primary RPE cells (*P* < 0.05, fold change >20). We used qRT-PCR for accurate confirmation of the expression of these genes in the RPE cells and hiPSCs. In particular, the expression level of *TNNT1* was equally high in all immortalized cell lines, while equally low in all three lots of primary cells. Furthermore, *TNNT1* mRNA was highly expressed in hiPSCs that can self-renew and the other two immortalized RPE cell lines, hTERT RPE-1 cells and ABM-RPE cells (Fig. 2 and Supplementary Fig. 1).

**Figure 3:**
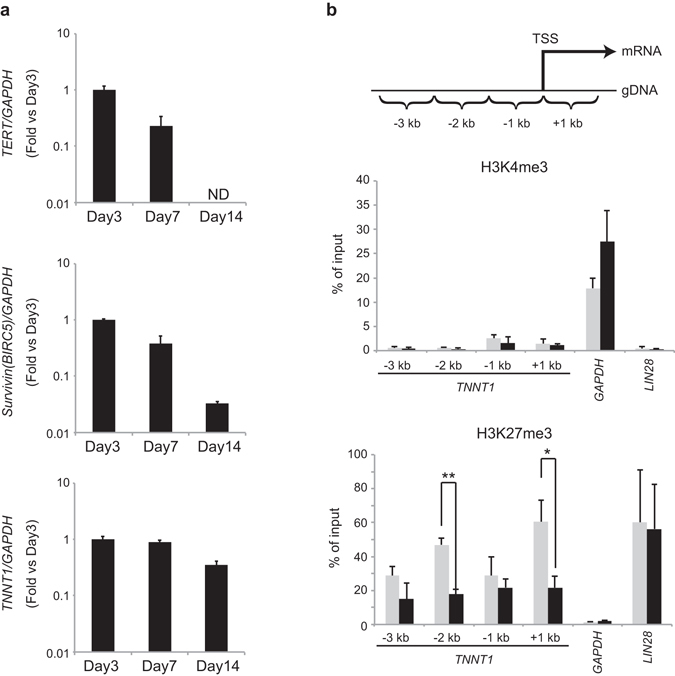
Time course analysis of *TNNT1* mRNA expression and ChIP-qPCR assay of the transcription start site of *TNNT1*. **(a)** Time course of the expression of *TERT*, *Survivin/BIRC5*, and *TNNT1* mRNA in primary RPE cells during culture for 3, 7 and 14 days as analyzed by qRT-PCR. Bar graph represents fold gene expression relative to day 3. **(b)** ChIP-qPCR assay of H3K4me3 and H3K27me3 in −3 kb, −2 kb, −1 kb, and + 1 kb regions from the TSS of *TNNT1*. Positive control experiments were performed with promoter regions of *GAPDH* (H3K4me3) and *LIN28* (H3K27me3). Results are means ± standard deviations of three independent experiments (black; ARPE-19 cells, gray; primary RPE cells). ND: not detected. *P < 0.05, **P < 0.01, Student’s *t*-test (n = 3).

Masutomi *et al*. have previously reported that the expression of *TERT* corresponds temporally with the entrance and transit of a large percentage of these synchronized cells through S phase in normal human cells^28^. To determine the schedule of *TNNT1* as well as *TERT* expression throughout the cell cycle, we monitored mRNA levels of *TNNT1* in primary RPE cells during cell growth. With increasing culture time, both the population of S-phase cells and the expression of *TERT* and *Survivin/BIRC5* decreased. In contrast, *TNNT1* was constitutively expressed even after 14 days of culture (Fig. 3a and Table 1). Thus, transcripts of *TNNT1* corresponded less well with the cell cycle state in primary RPE cells than did those of *TERT* and *Survivin/BIRC5*. These results suggested that *TNNT1* is a stable marker gene of immortalized RPE cells without variable background signal derived from the cell-cycle state of normal RPE cells.

**Table 1:** Cell cycle analysis of primary RPE cells.

Day	G_0_/G_1_ (%)	S (%)	G_2_/M (%)
3	73.0	16.1	8.1
7	85.6	4.7	7.5
14	92.7	2.8	4.8

To ascertain whether the upregulation of *TNNT1* expression in immortalized RPE cells was due to epigenetic regulation, we explored the chromatin status at the *TNNT1* locus in immortalized RPE cells. We performed ChIP-qPCR in primary RPE and ARPE-19 cells using antibodies specific for trimethylated lysine 27 of histone H3 (H3K27me3), a repressive mark, and trimethylated lysine 4 of histone H3 (H3K4me3), an active mark, to determine their mark in −3 kb, −2 kb, −1 kb and +1 kb regions from the transcription start site (TSS) of *TNNT1*. H3K27me3 was significantly reduced at −2 kb and +1 kb regions relative to the TSS in ARPE-19 cells when compared to primary RPE cells. On the other hand, we did not recognize any appreciable difference in H3K4me3 enrichment at the *TNNT1* gene locus between primary RPE cells and ARPE-19 cells (Fig. 3b). These findings implied that loss of H3K27me3 mark at the *TNNT1* locus promotes transcriptional activation of *TNNT1*.

### Detection of Immortalized RPE Cells Using qRT-PCR

**Figure 4:**
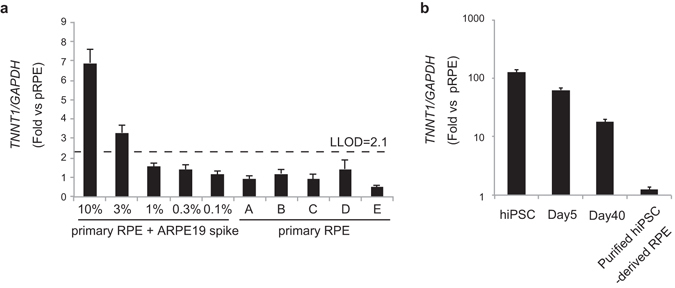
Validation of the *TNNT1* qRT-PCR assay. **(a)** qRT-PCR analysis of *TNNT1* mRNA expression in primary RPE cells spiked with different amounts of ARPE19 cells and 5 lots of primary RPE cells. The LLOD (dashed line) was determined as described in the Results section. **(b)** Time course of the expression of *TNNT1* mRNA during differentiation of hiPSCs to RPE cells. All values are expressed as mRNA levels relative to the mean of 5 lots of primary RPE cells. Results are means ± standard deviations (n = 3).

To develop and validate a qRT-PCR assay detecting *TNNT1* mRNA, we spiked 5 × 10^4^ (10%), 1.5 × 10^4^ (3%), 5 × 10^3^ (1%), 1.5 × 10^3^ (0.3%), and 5 × 10^2^ (0.1%) ARPE-19 cells into 5 × 10^5^ primary RPE cells. Total RNA was extracted from the mixtures and subjected to qRT-PCR using *TNNT1-*specific primers. Relative values of *TNNT1* mRNA of the 10%, 3%, 1%, 0.3%, and 0.1% ARPE-19 cell-spiked samples were 6.9 ± 0.7, 3.3 ± 0.4, 1.5 ± 0.2, 1.4 ± 0.3, and 1.2 ± 0.1 (ratio to the average level of primary RPE cells ± standard deviation), respectively. Correspondingly, to determine the lower limit of detection (LLOD) of ARPE-19 cells contained in primary RPE cells, relative values of *TNNT1* mRNA were analyzed in five lots of primary RPE cells. The LLOD of the assay signal is commonly calculated as the mean plus 3.3-fold the standard deviation of the measurement of negative controls^29^. Based on the dispersion of signals from five lots of primary RPE cells as a negative control (1 ± 0.35 [fold over the average of five lots ± standard deviation]), the LLOD of the *TNNT1* qRT-PCR assay was calculated as 2.1 (Fig. 4a). These results indicated that the *TNNT1* qRT-PCR assay is able to detect 3% of ARPE-19 cells contained in primary RPE cells. Next, RNA isolated from undifferentiated hiPSCs, hiPSCs after 5 and 40 days of differentiation, and purified hiPSC-derived RPE cells was subjected to the *TNNT1* qRT-PCR assay. The mRNA expression of *TNNT1* gradually decreased during differentiation to a final level below the LLOD. Relative values of *TNNT1* mRNA of the hiPSC, day 5, day 40, and purified hiPSC-derived RPE samples were 123.8 ± 18.0, 62.8 ± 6.0, 17.7 ± 2.2, and 1.2 ± 0.1, respectively (Fig. 4b). These results indicated that *TNNT1* mRNA levels are strongly dependent on the extent of residual undifferentiated hiPSCs in differentiated RPE cells.

### Expression of *TNNT1* in Various Cancer Tissues

**Figure 5:**
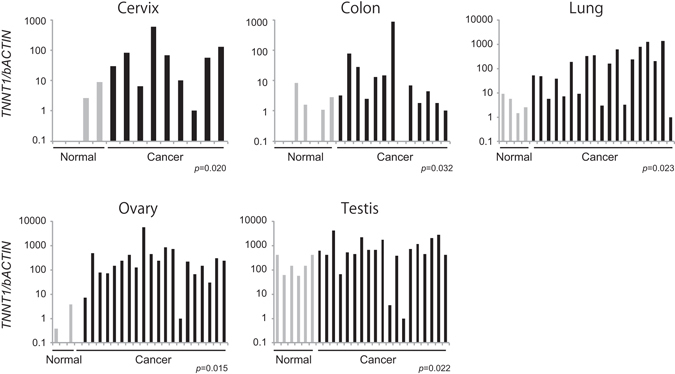
Versatility of *TNNT1* as a marker of immortalized cells. Expression of *TNNT1* in human cancer tissues and normal tissues was quantified using the TissueScan Cancer Survey Panel. Data were obtained using the comparative CT method, with values normalized to β-actin levels. Bar graph represents fold expression of *TNNT1* relative to the lowest detected sample in cancer tissue samples (black; cancer tissue, gray; normal tissue). Blank: not detected. Statistical analysis was performed using the Mann–Whitney U test.

To investigate the versatility of *TNNT1* as a marker of immortalized cells, we examined *TNNT1* expression levels in various types of human cancer tissues and normal tissues using a TissueScan array spotted with cDNAs derived from 18 different cancer or normal tissues (adrenal glands, breasts, cervix, colon, endometrium, esophagus, kidneys, liver, lungs, lymphoid tissue, ovaries, pancreas, prostate, stomach, testes, thyroid gland, urinary bladder, and uterus). The tissues displaying a statistically significant difference in *TNNT1* expression are shown in Fig. 5, other tissues are shown in Supplementary Fig. 2. As shown in Fig. 5, cancer tissues of cervix, colon, lungs, ovaries, and testes showed significantly elevated levels of *TNNT1* expression as compared with the corresponding normal tissues. As for ovarian cancer, these results agree with a study by Bapat *et al*. showing a significant upregulation of *TNNT1* expression in ovarian cancer in *in silico* transcriptomics analysis^30^.

### Overexpression of *TNNT1* Enhances Cell Migration and Actin Polymerization

**Figure 6:**
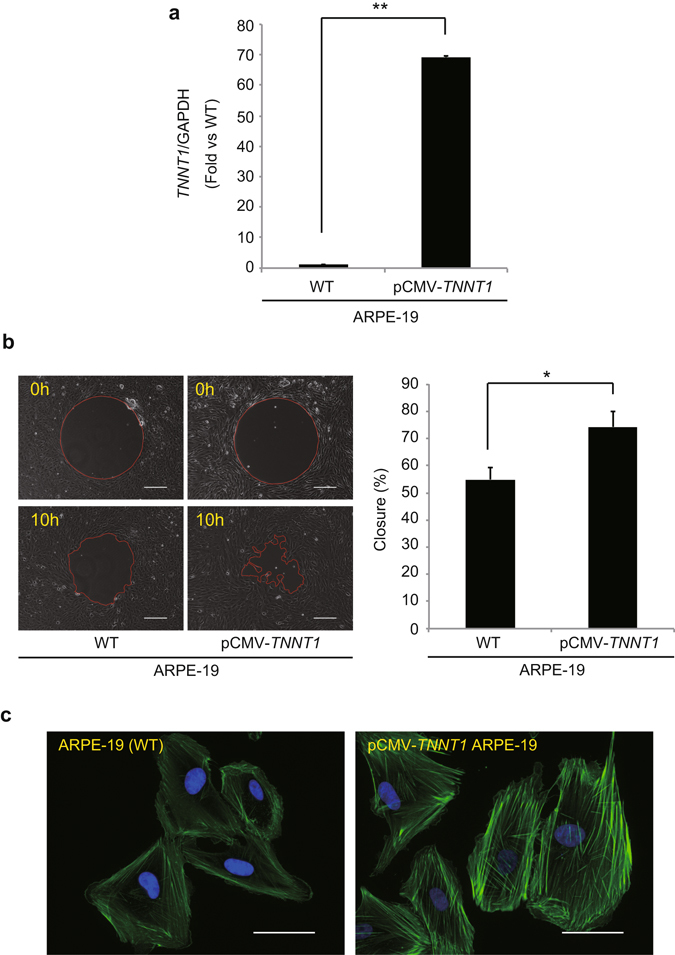
Overexpression of *TNNT1* enhances cell migration and actin polymerization. **(a)** qRT-PCR analysis of *TNNT1* mRNA in ARPE-19 cells (WT) and pCMV-*TNNT1* ARPE-19 cells (pCMV-*TNNT1*). Bar graph represents fold expression of *TNNT1* relative to ARPE-19 cells. **(b)** Phase-contrast images of migration assay of ARPE-19 cells (WT) and pCMV-*TNNT1* ARPE-19 cells (pCMV-*TNNT1*). Scale bar: 200 µm. Closure rates were calculated as described as material methods. **(c)** Immunocytochemistry of phalloidin-Alexa 488 of ARPE-19 and pCMV-*TNNT1* ARPE-19 cells. Scale bar: 50 µm. Results are means ± standard deviations of three independent experiments. *P < 0.05, **P < 0.01, Student’s *t*-test (n = 3).

On the basis of the above findings, we predicted that *TNNT1* might play a role in immortalization. To investigate this, we first aimed to stably transduce the *TNNT1* gene into primary RPE cells via transgene integration into a zinc finger nuclease (ZFN)-targeting site using a lentiviral vector delivery. However, we were not able to generate primary RPE cells overexpressing *TNNT1* that were resistant to puromycin. Instead, we established ARPE-19 cells overexpressing *TNNT1* by inserting *TNNT1* at an AAVS1 site cleaved with ZFN (pCMV-*TNNT1* ARPE-19). As expected, the pCMV-*TNNT1* ARPE-19 cells showed an approximately 70-fold increase in *TNNT1* mRNA (Fig. 6a). As expression of *TERT*, a key player in telomere maintenance in immortalized cells, is involved in the migration of HCT116 cells^31^, we examined the effects of *TNNT1* on cell migration by a gap closure migration assay. The pCMV-*TNNT1* ARPE-19 cells showed significantly higher inward migration than wild-type ARPE-19 cells (Fig. 6b). Complementary experiments demonstrated that transient expression of *TNNT1* in hTERT RPE-1 cells also enhanced their migration and supported our results (Supplementary Fig. 3). Conversely, shRNA-mediated *TNNT1* knockdown in ARPE-19 cells significantly suppressed migration of knockdown cells (Supplementary Fig. 4). Moreover, we observed a distinct enhancement of actin polymerization induced by overexpression of *TNNT1* in ARPE-19 cells (Fig. 6c). These results suggested that *TNNT1* expression affects actin filament organization and enhances cell migration ability in immortalized RPE cells.

## Discussion

Tumorigenicity, which is attributed to residual undifferentiated cells and transformation of cells, is one of the major concerns in developing hESC/hiPSC-derived CTPs. Here, we introduced a novel *in vitro* assay for detecting immortalized RPE cells and showed its efficacy using hiPSC-derived RPE cells. Cellular immortalization is widely known as a key step in the development of most human cancers, and the importance of an immortalized marker is illustrated by the fact that four immortalized RPE cell lines spontaneously arose from primary human RPE cells during *in vitro* culture^20–23^.

Jiang *et al*. previously reported that *TERT*-expressing immortalized RPE cells do not form tumors in nude mice after subcutaneous transplantation^32^. In line with this report, we found that the soft agar colony formation assay failed to detect colonies of immortalized RPE cell lines. Contrary to our expectation, *TERT* and *Survivin/BIRC5*, which are highly expressed in various cancer cells, appeared not suitable as marker genes of immortalized RPE cells. Thus, such assays detecting transformed cells cannot be applied for detecting immortalized RPE cells. To develop an *in vitro* assay system detecting immortalized RPE cells, we comprehensively screened genes expressed in immortalized RPE cells and successfully identified 15 candidate marker genes, the expression of which was 20 times higher in immortalized than in primary RPE cells. Some promising genes have been reported to be related to cancer. *CAMK2N1* and *CDH11* have been shown to encode tumor suppressors^33, 34^. *CARD6* is significantly expressed in gastric, colorectal, and esophageal cancers^35^. Other promising genes include the pregnancy-specific glycoprotein family genes (*PSG1*, *PSG4*, and *PSG7*) and *BDNF*, *CLDN11, GAD1*, *IL15*, *PAPSS2*, *PLK2, SHOX2, TNNT1*, and *TWIST1*; these were previously identified without any known immortalization-associated functionalities. Among the 15 candidate genes, only *TNNT1* expression exhibited more than 100-fold increase in hiPSCs and immortalized RPE cell lines as compared to primary RPE cells. Therefore, we focused on *TNNT1* as an immortalized RPE cell marker and developed a qRT-PCR assay for *TNNT1* transcript detection. A validation study revealed that the *TNNT1* qRT-PCR assay can detect as few as 3% of ARPE-19 cells spiked into primary RPE cells.

H3K27me3 but not H3K4me3 was strongly enriched in the *TNNT1* gene locus in primary RPE cells than in ARPE-19 cells, indicating that *TNNT1* expression of normal RPE cells was possibly repressed under epigenetic control. The loss of H3K27me3 at the *TNNT1* locus could explain the marked difference in its expression between ARPE-19 and primary RPE cells. However, it is still unknown whether loss of the H3K27me3 mark in the *TNNT1* gene locus occurs in the process of cell immortalization.

The troponin complex forms the Ca^2+^-sensitive molecular switch that regulates striated muscle contraction in response to alterations in the intracellular calcium concentration. Troponin T binds tropomyosin to anchor the troponin complex to a specific location of thin filaments. In vertebrates, three homologous genes have evolved to encode three muscle type-specific troponin T isoforms. *TNNT1* is known to be a slow skeletal muscle-specific troponin T^36^. shRNA-mediated *TNNT1* knockdown did not affect the immortalization of ARPE-19 cells even if cultured for more than 10 passages (Kuroda *et al*., unpublished observation). However, in the current study, we found that *TNNT1* overexpression increased migration and enhanced actin polymerization in ARPE-19 cells. We also repeated migration assay with hTERT RPE-1 transiently expressing *TNNT1* and obtained the similar results that *TNNT1* enhanced migration of its overexpressed cells. As microinjection of tropomyosin into epithelial cells has been reported to induce rapid cell migration^37^, *TNNT1* likely contributes to the interaction of actin and tropomyosin, which regulates cell migration and invasion^38^. In the context of cancer, we demonstrated substantial expression of *TNNT1* in cancer tissues of the cervix, colon, lungs, ovaries, and testes. Similarly, Gu *et al*. reported that postoperative relapse of gallbladder carcinoma is positively related to *TNNT1* expression^39^. According to our results and previous reports, *TNNT1* seems to play multiple roles in cancer. *TNNT1* may partly but not essentially function in cell immortalization, the mechanism of which remains to be uncovered.

Tumorigenicity, which is attributed to residual undifferentiated cells and transformation of cells, is one of the major concerns in the development of hESC/hiPSC-derived CTPs. Here, we introduced a novel *in vitro* assay for detecting immortalized RPE cells and showed its efficacy in hiPSC-derived RPE cells. Our *TNNT1* qRT-PCR assay is rapid and simple, and will hopefully contribute to process validation and quality control of hPSC-derived CTPs in future.

## Materials and Methods

### Cell Culture

Human fetal RPE cells were obtained from Lonza (lot. A, lot-0F3292; lot. B, lot-0F3237; lot. C, lot-0000182939; lot. E, lot-0000249852) or ScienCell Research Laboratories (lot.D, 6011) and maintained in Retinal Pigment Epithelial Cell Basal Medium (Lonza) containing supplements (L-glutamine, GA-1000, and bFGF; Lonza). ARPE-19 and ARPE-19/HPV-16 cells were obtained from ATCC and maintained in DMEM/F12 containing 20% fetal bovine serum (FBS) and 1% penicillin/streptomycin (all from Gibco). h1RPE7 cells were obtained from ECACC and maintained in Ham’s F-10 medium containing 20% FBS, 1 μg/mL puromycin, and 1% penicillin/streptomycin. hTERT RPE-1 cells were obtained from ATCC and maintained in DMEM/F12 containing 10% FBS and 1% penicillin/streptomycin. ABM-RPE cells were obtained from Applied Biological Materials (ABM) Inc. and maintained in Prigrow medium (ABM) containing 10% FBS and 1% penicillin/streptomycin. The hiPSC line 201B7 was obtained from RIKEN and maintained in mTeSR1 medium (STEMCELL Technologies) on Matrigel-coated dishes. Differentiation of 201B7 into RPE cells was stimulated as previously described^13^. HeLa cells were obtained from ATCC and maintained in DMEM containing 10% FBS and 1% penicillin/streptomycin. All cells were cultured at 37 °C in a humidified atmosphere of 5% CO_2_ and 95% air.

### Soft Agar Colony Formation Assay

A soft agar colony formation assay was carried out using the CytoSelect 96-well Cell Transformation Assay kit (Cell Biolabs) as described previously^13^. Cells were cultured for 30 days. Colonies were lysed and quantified with CyQuant GR dye using a fluorometer equipped with a 485/520 nm filter set (Wallac 1420 ARVOsx multilabel counter, PerkinElmer).

### qRT-PCR

Total RNA was isolated using RNeasy Mini Kit (Qiagen) and treated with DNase I according to the manufacturer’s instructions. qRT-PCR was performed with the QuantiTect Probe RT-PCR Kit (Qiagen) on a 7300 Real-Time PCR System (Applied Biosystems). Target gene expression was normalized to GAPDH mRNA, which was quantified using TaqMan human GAPDH control reagents (Applied Biosystems). Ct values of greater than 35 were regarded as “not detected” (“N.D.”). The sequences of the primers and probes used in the present study are listed in Table S1. Oligonucleotides were obtained from Sigma-Aldrich.

### GeneChip and Biostatistical Analysis

Total RNA was isolated from three lots of primary RPE cells and three immortalized RPE cell lines using an RNeasy Mini Kit (Qiagen) and treated with DNase I according to the manufacturer’s instructions. Samples were converted into biotinylated cRNA using Two-Cycle Target Labeling and Control Reagents (Affymetrix). Labeled RNA was processed for microarray hybridization to Human Genome U133 Plus 2.0 GeneChips (Affymetrix). Five samples of each RPE cell strain were used in this experiment. An Affymetrix GeneChip Fluidics Station was used to perform streptavidin/phycoerythrin staining. The hybridization signals on the microarray were scanned using a GeneChip Scanner 3000 (Affymetrix) and analyzed using Expression console software (Affymetrix). Normalization was done by global scaling with the arrays scaled to a trimmed average intensity of 500 after excluding the 2% of probe sets with the highest and the lowest values. The National Center for Biotechnology Information Gene Expression Omnibus (NCBI GEO) accession number for the microarray data is GSE80985. Informational probe sets were filtered from the data set in three steps. First, probe sets were regarded as “present” when indicated as “present” by “absolute analysis” using Expression console software in all five samples from one strain. Probe sets regarded as “absent” in more than one immortalized RPE line were eliminated. Then, probe sets for which no significant difference was observed among the cell lines by ANOVA (*P* < 0.05) were eliminated. Finally, probe sets for which the difference between the maximum and minimum mean values of probe sets in the cell lines was equal to or more than 20-fold were used for further analysis.

### Cell Cycle Analysis

Cell cycle analysis was performed by fluorescence-based cell sorting as previously described^40^. Briefly, after collection, the cells were fixed with 70% ethanol for 2 h at 4 °C, and were treated with 0.25 mg/mL RNase solution for 30 min at 4 °C and stained with a PI solution (50 μg/mL), and analyzed on a FACS Calibur (BD). Cell cycle distribution was analyzed with the FlowJo cell cycle Watson (Pragmatic) model.

### ChIP-qPCR Assay

Primary RPE cells and ARPE-19 cells were treated with trypsin/EDTA (0.25% trypsin, 0.5 mM EDTA in phosphate-buffered saline (PBS) for 5 min at room temperature. Dissociated cells were treated with 1% paraformaldehyde for 8 min at room temperature. After quenching the PFA crosslinking reaction with 125 mM glycine, the fixed cells were washed with PBS. The cells were suspended in lysis buffer (50 mM Tris-HCl (pH 8), 10 mM EDTA, 1% SDS, and Complete protease inhibitor cocktail (Roche)) and sonicated to an average fragment size of 200–1000 bp. Solubilized chromatin was diluted with ChIP dilution buffer (16.7 mM Tris-HCl (pH 8.1), 167 mM NaCl, 1.2 mM EDTA, 0.01% SDS, 1% Triton X-100 and Complete protease inhibitor cocktail) and clarified by centrifugation at 13,000 × *g* for 10 min at 4 °C. The supernatant was pre-cleared with protein G-Dynabeads (Life Technologies), which were pre-blocked with rabbit control IgG (ab46540, Abcam) at 4 °C for 1 h. The pre-cleared chromatin was incubated with anti-H3K4me3 (ab8580, Abcam) and anti-H3K27me3 (17–622, Merck Millipore) antibodies overnight at 4 °C. Immune complexes were bound to protein G-Dynabeads for 2 h at 4 °C. The beads were sequentially washed with low-salt buffer (20 mM Tris-HCl (pH 8.1), 150 mM NaCl, 2 mM EDTA, 0.1% SDS and 1% Triton X-100), high-salt buffer (20 mM Tris-HCl (pH 8.1), 500 mM NaCl, 2 mM EDTA, 0.1% SDS and 1% Triton X-100), LiCl wash buffer (10 mM Tris-HCl (pH 8.1), 250 mM LiCl, 1 mM EDTA, 1% IGEPAL-CA630, 1% Na deoxycholate), and TE buffer (10 mM Tris-HCl (pH 8.0), 1 mM EDTA (pH 8.0)). Immune complexes bound to protein G-Dynabeads were suspended in Complete elution buffer (20 mM Tris-HCl (pH 7.5), 5 mM EDTA, 50 mM NaCl, 1% SDS, and 50 μg/mL proteinase K) and incubated for 2 h at 68 °C. After incubation, DNA was purified with ChIP DNA Clean & Concentrator Kit (Zymo Research) and analyzed by qPCR using the following gene-specific primers: *LIN28* forward, GGGTTGGGTCATTGTCTTTTAG; reverse, AAAGGGTTGGTTCGGAGAAG, *GAPDH* forward, CGGGATTBTCTGCCCTAATTAT; reverse, GCACGGAAGGTCACGATGT, *TNNT1* −3 kb, GPH1021012 (-)03 A; *TNNT1* −2 kb, GPH1021012 (−)02 A; *TNNT1* −1 kb, GPH1021012 (−)01 A; *TNNT1* +1 kb, GPH1021012 (+)01 A. The piTect ChIP qPCR primers GPH1021012 (+)01 A, (−)01 A, (−)02 A, and (−)03 A were obtained from Qiagen. qPCR data were normalized to 5% of the purified input DNA, which was used as a measure of the total amount of chromatin present in the sample.

### TissueScan™ Cancer and Normal Tissue cDNA Array Analysis

The TissueScan Cancer Survey Panel 4 × 96-III (Origene) was used according to the manufacturer’s protocol. The panel consisted of 381 cDNA samples covering 18 different cancers. β-Actin expression was used to normalize relative *TNNT1* expression in tissue samples and was quantified using TaqMan human β-actin control reagents (Applied Biosystems). For statistical analysis, we regarded not detected as zero. Some samples were excluded from following statistical analysis, because the number of samples contained in one group was less than three (breast, endometrium, bladder, and uterus).

### Generation of a *TNNT1*-overexpressing ARPE-19 Cell Line

For *TNNT1* overexpression, a transgene encoding *TNNT1* was inserted into the AAVS1 locus in the ARPE-19 cell using the CompoZr Targeted Integration-AAVS1 kit (Sigma-Aldrich) per the manufacturer’s protocol. The base sequence of pCMV promoter and human *TNNT1* ORF (NM_001126133) was *de novo* synthesized (GenScript) and cloned into pZDonor-AAVS1 Puromycin Vector (Sigma-Aldrich). ARPE-19 cells were transduced with 30 μg targeting vector and 5 μL zinc finger nuclease (ZFN) mRNA using a Nepa21 electroporator (Nepa Gene). Then, the cells were treated with 2–5 μg/mL puromycin for 1 day. *TNNT1* mRNA expression was confirmed by qRT-PCR.

### Transient transfection of *TNNT1*

For transient expression of *TNNT1*, hTERT PRE-1 cells plated in 12-well dishes were transiently transfected with pCMV-*TNNT1* (RG225325, ORIGENE) or pCMV-Entry vector (PS100001, ORIGENE) using TransIT-X2 Reagent (Mirus Bio LLC) according to the manufacturer’s instructions. The total amount of DNA was adjusted to 1 μg. Twenty-four hours after transfection, cells were cultured for 2 days in normal media, and then subjected to migration assay.

### Lentivirus-derived RNAi and generation of *TNNT1* knockdown cell line

ARPE-19 *TNNT1*-knockdown cells were generating by transducing ARPE-19 with lentivirus particles that expressed a *TNNT1*-targeting set of shRNA. Briefly, Lenti-X 293 T cells were transfected with individual clones from Sigma MISSION shRNA targeting set (TRCN0000118897) or the control shRNA plasmid along with a MISSION lentivirus packaging Mix (Sigma-Aldrich) according to the manufacture’s procedures. Media containing viruses were collected 48 hours after transfection. ARPE-19 cells were transduced with the viruses in the presence of 8 µg/ml polybrene (Sigma-Aldrich) for 24 hours, and then subjected to selection with 2 µg/ml puromycin for 48 hours.

### Migration Assay

Cell migration was measured using a Radius 24-Well Cell Migration Assay Kit (Cell Biolabs) according to the manufacturer’s instructions. Each well contains a circular 680-μm-diameter “gel spot” to which cells do not attach. Before the experiment, cells were cultured to confluence. At the start of the experiment, the gel spot was removed, after which cells gradually populated the circular void space. After a 10-h incubation at 37 °C, migrated cells were captured using phase-contrast microscopy and the migration of cells into the initially cell-free area was analyzed using Keyence image analysis software.

### Actin Filament Staining

Cells were fixed with 4% paraformaldehyde in PBS for 20 min at room temperature. After washing with PBS, the cells were permeabilized with 0.2% Triton-X100 in PBS for 15 min and blocked with 2% bovine serum albumin in PBS for 30 min. Samples were incubated for 20 min with 5 units/mL of Alexa Fluor 488 phalloidin (A12379, Thermo Fisher). Nuclei were stained with 10 μg/mL DAPI (Invitrogen) and examined under a BZ-X710 fluorescence microscope (Keyence).

### Statistical Analysis

Statistical analysis was performed using Systat 13 Software (Systat Software Inc., CA). Comparisons were made using the Student *t*-test (for two groups) and the Mann–Whitney U test (for normal vs. cancer tissues, Fig. 5 and Supplementary Fig. 1). *P*-values < 0.05 were considered significant.

## Electronic supplementary material

Supplementary information
