# Supplementary material for: Identification of a Gene Encoding Slow Skeletal Muscle Troponin T as a Novel Marker for Immortalization of Retinal Pigment Epithelial Cells
Source: Sci Rep. 2017 Aug 15;7:8163. doi: 10.1038/s41598-017-08014-w (PMC5557831; doi:10.1038/s41598-017-08014-w)
Supplement: Supplementary file 1 — Supplementary information [file 41598_2017_8014_MOESM1_ESM.pdf]

Identification of a Gene Encoding Slow Skeletal Muscle Troponin T as a Novel Marker for Immortalization of Retinal Pigment Epithelial Cells

Takuya Kuroda[1, 2], Satoshi Yasuda[1, 2], Hiroyuki Nakashima[1, 3], Nozomi Takada[1, 4], Satoko Matsuyama[1, 4], Shinji Kusakawa[1, 2], Akihiro Umezawa[3], Akifumi Matsuyama[4], Shin Kawamata[2], Yoji Sato[1, 2, 5, 6, 7*]

[1]Division of Cell-Based Therapeutic Products, National Institute of Health Sciences, Tokyo, Japan

[2]Foundation for Biomedical Research and Innovation, Kobe, Japan

[3]Department of Reproductive Biology, National Research Institute for Child Health and Development, Tokyo, Japan

[4]Platform of Therapeutics for Rare Disease, National Institutes of Biomedical Innovation, Health and Nutrition, Osaka, Japan

[5]Department of Quality Assurance Science for Pharmaceuticals, Graduate School of Pharmaceutical Sciences, Nagoya City University, Nagoya, Japan

[6]Department of Cellular & Gene Therapy Products, Graduate School of Pharmaceutical Sciences, Osaka University, Osaka, Japan

[7]Department of Drug Discovery and Evolution, Graduated School of Pharmaceutical Sciences, Kyushu University, Fukuoka, Japan

[*]Corresponding Author: Yoji Sato. Mailing address: Division of Cell-based Therapeutic Products, National Institute of Health Sciences, 1-18-1, Kamiyoga, Setagaya-ku, Tokyo 158-8501, Japan. Phone: 81-3-3700-9373. Fax: 81-3-3700-9373. E-mail: yoji@nihs.go.jp

Supplementary Information

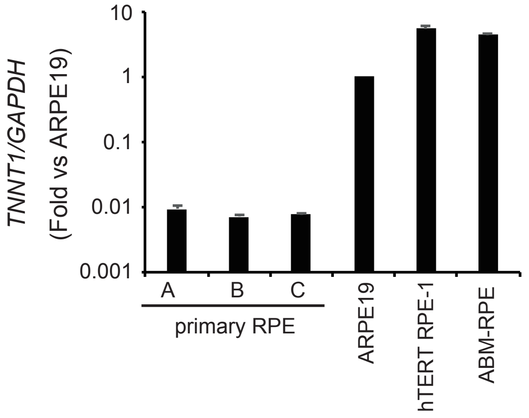

Supplementary Figure 1. qRT-PCR analysis of *TNNT1* in immortalized RPE cell lines (hTERT RPE-1 and ABM-RPE). Bar graph represents fold gene expression relative to ARPE-19 cells. Results are means ± standard deviations (n = 3).

Supplementary Figure 2

Kuroda et al

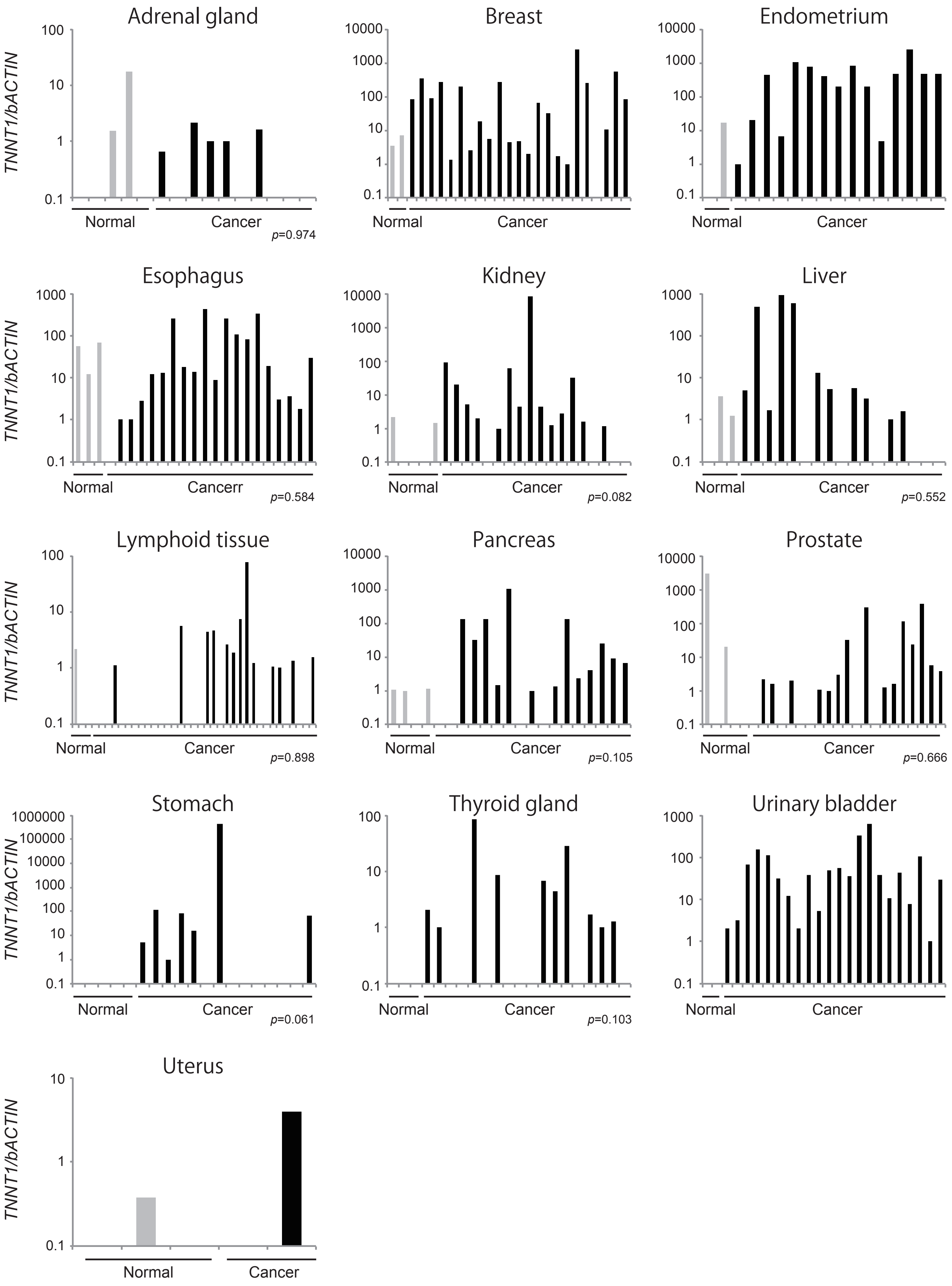

Supplementary Figure 2. The expression of *TNNT1* in various cancer tissues. Expression profiles of *TNNT1* in human cancer tissues and normal tissues were quantified using the TissueScan Cancer Survey Panel. Data were obtained using the comparative CT method with the values normalized to ß-actin levels. Bar graphs represented the fold expression of *TNNT1* relative to the lowest detected sample in cancer tissue samples (black; cancer tissue, gray; normal tissue). Blank: not detected. Statistical analyses of breast, endometrium, urinary bladder and uterus were not performed, because the number of the samples included in one group was less than three.

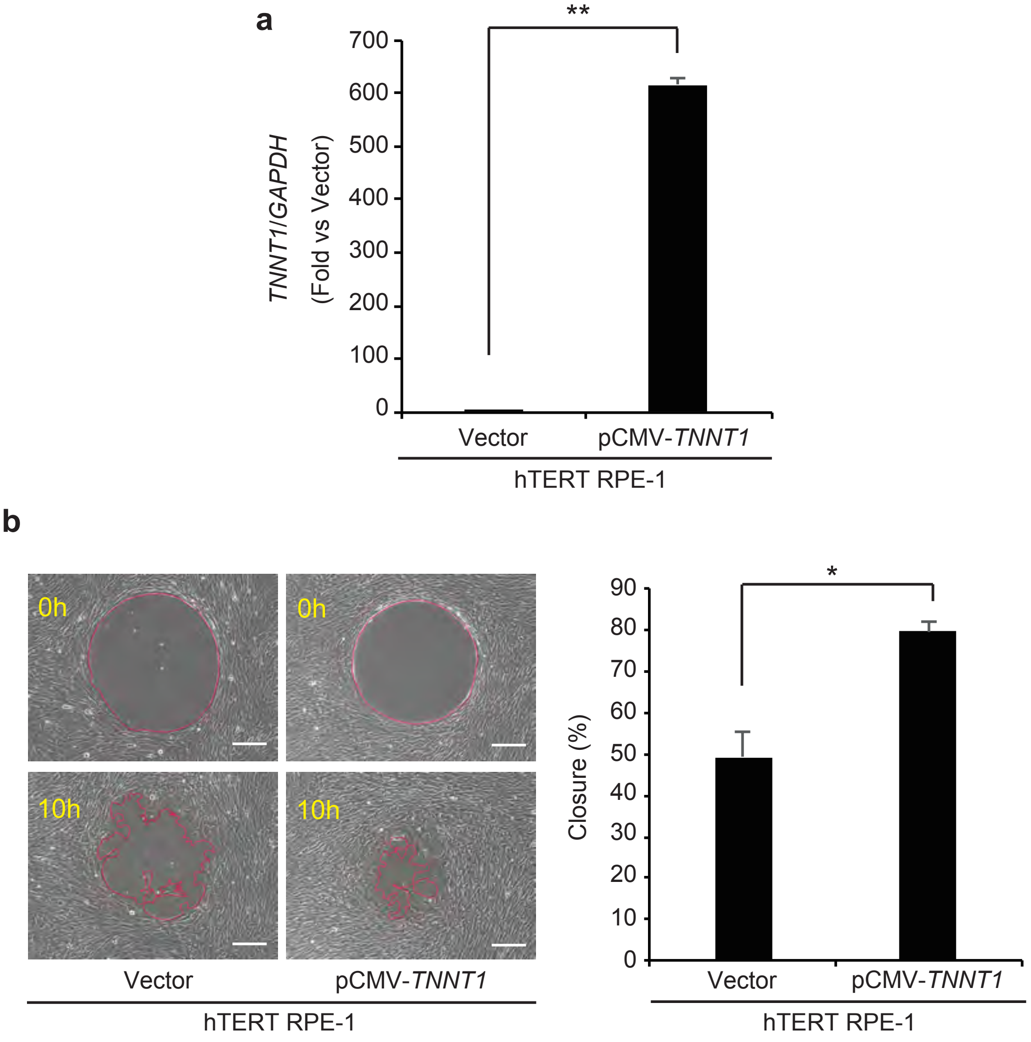

Supplementary Figure 3. Overexpression of *TNNT1* enhances cell migration of hTERT RPE-1 cells. (a) qRT-PCR analysis of *TNNT1* mRNA in hTERT RPE-1 cells (Vector) and pCMV-*TNNT1* hTERT RPE-1 cells (pCMV-*TNNT1*). Bar graph represents fold expression of *TNNT1* relative to sample of vector. (b) Phase-contrast images of migration assay of hTERT RPE1 cells (Vector) and pCMV-*TNNT1* hTERT RPE1 cells (pCMV-*TNNT1*). Scale bar: 200 µm. Closure rates were calculated as described as material methods. Results are means ± standard deviations (n = 3). *$P < 0.05$, **$P < 0.001$, Student' s t-test (n = 3)

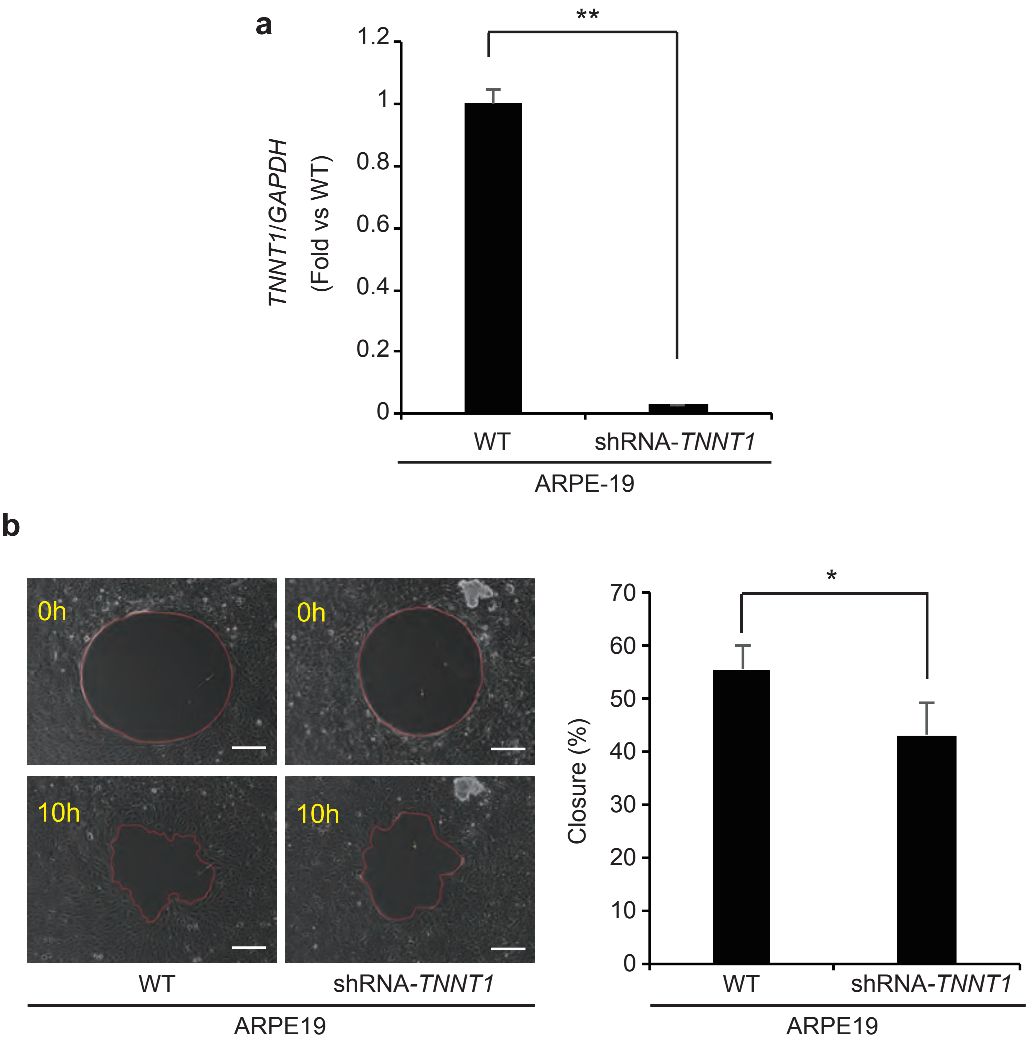

Supplementary Figure 4. Knockdown of *TNNT1* suppresses cell migration of ARPE19 cells. (A) qRT-PCR analysis of *TNNT1* mRNA in ARPE-19 cells (WT) and shRNA-*TNNT1* ARPE-19 cells (shRNA-*TNNT1*). Bar graph represents fold expression of *TNNT1* relative to ARPE-19 cells. (B) Phase-contrast images of migration assay of ARPE-19 cells (WT) and shRNA-*TNNT1* ARPE-19 cells (shRNA-*TNNT1*). Scale bar: 200 μm. Closure rates were calculated as described as material methods. *$P < 0.05$, **$P < 0.001$, Student' s t-test (n = 3)

Supplementary Table 1. Probe and primer sequences

Gene	Probe sequences (5' →3')	Forward primer sequences (5'→3')	Reverse primer sequences (5'→3')
TNNT1	ATCCTCTCCGAGCGTAAGAAGCCTCTGG	GGGGCGGGAGATGAAGGTG	GGTCGAACTTCTCAGACTCCAG
BDNF	TTTCAAGGACTGTGACCGTCCCGCCC	GTGTGTGACAGTATTAGTGAGTGG	CTCGTAGAAGTATTGCTTCAGTTGG
CAMK2N1	TATGACCGACAAGGCACCTCCTGGTG	CGGAGCAAGCGGGTTGTTA	CTCTCCCTTAACTCATTGTCTTTGG
CARD6	ACCAAGTTATGAGGGATCAGAAACCAGCCT	CTTCGAGAATTCAGAAACCACAGAG	CCATGTTCAAACATAACAGGACATC
CDH11	TGCCAAAGACCCTGATGCTGCCAACA	CACCGTGGTTGGGAGAGTG	TGTCGAGGTCAGTGTGACGA
CLDN11	CCTTGTTGCCACCATCTGGTTCCCTGTG	CTGGCTGGTGTTTTGCTCATTC	TGGTGGTCTCACGGTGGG
GAD1	CGAGGATGACCTGTGCGAACCCCATACT	GGAGCAGATCCTGGTTGACTG	GCCAATAATATCCAATCCAGTGGAG
IL15	TCCTCCAGTTCCTCACATTCTTTGCATCCA	CATCCTAGCAAACAACAGTTTGTC	TGGACAATATGTACAAAACTCTGCA
PAPSS2	TGGACCAATACCGTCTGACACCTCTGGA	TGCTGGAGAAAATAAGATGGAATGA	CGCAACTGGAATGCAAACACC
PLK2	TGCTACAGCTGCCAAGAGTCCCTCTGAC	CACCACAGTTGCCAGGTCTG	GGTACTGTCTTCAAGGCATTCAC
PGS1	AGGTGAAACGTCCAGTTACTCCTCTAGTCC	ACACCTTACACATCATAAAGGGAGA	GGCTTAGGAGTCTCCAGGTG
PGS4	CGAGTTGTCCACCTCCAGCTTATAGGGC	AGCTCCAAATCCATCACAGTCAA	GCTCACATGTCAGGTACAAGGG
PGS7	AGCTCCAAATCCGTGACAGTCAGAGTCTCT	GCTTGCTCTGTTCGTAACTCAG	GAGTGGGTCTTGCTCTTTGAGG
SHOX2	CAGTCGCTGGCTCAGTTCCTCTCGCA	ACGAGACCCACTATCCCGAC	CTTCGATTTTGAAACCAAACCTGC
TWIST1	TCCAGCTCCAGAGTCTCTAGACTGTCCATT	GGCCGGAGACCTAGATGTCA	GGATTTTGCTCTTCTAATTTCCAAGA

TERT	CAGCCTCCAGACGGTGTGCACCAAC	CCTGTTTCTGGATTTGCAGGTG	GCACACATGCGTGAAACCTG
Survivin	CCTCGGCCATCCGCTCCGGG	ACCACCGCATCTCTACATTCAAG	CTCAGTGGGGCAGTGGATGA
